# Surgical Treatment of SplenicFlexure Colon Cancer: Analysisof Short-Term and Long-Term Outcomes of Three DifferentSurgical Procedures

**DOI:** 10.3389/fonc.2022.884484

**Published:** 2022-06-24

**Authors:** Mingjin Huang, Xiaojie Wang, Yu Shao, Shenghui Huang, Ying Huang, Pan Chi

**Affiliations:** ^1^ Department of Colorectal Surgery, Fujian Medical University Union Hospital, Fuzhou, China; ^2^ Department of Pathology, Fujian Medical University Union Hospital, Fuzhou, China

**Keywords:** splenic flexure cancer, surgical procedure, lymph node metastasis, postoperative outcome, oncological outcome

## Abstract

**Background:**

The aim of this study was to compare the short- and long-term outcomes of splenic flexure colectomy (SFC), left hemicolectomy (LHC) and extended left hemicolectomy (ELHC) for splenic flexure colon cancer.

**Methods:**

Between January 2011 and December 2018, 117 patients with splenic flexure cancer were enrolled in the study. We retrospectively compared the postoperative, pathological and long-term outcomes of patients with splenic flexure cancer.

**Results:**

Of the 117 patients, 73 (62.4%) underwent SFC, 22 (18.8%) underwent LHC, and 22 (18.8%) underwent ELHC. No statistically significant differences were found among the groups regarding postoperative complications, pathological data or recurrence. No metastatic lymph nodes at the root of the inferior mesenteric artery (IMA) were observed; lymph node metastasis appeared at the root of the middle colic artery (MCA), but in a low proportion of cases (4.4%). Looking at long-term prognosis, no differences were observed among the three groups regarding both 5-year overall survival (94.0% vs 90.2% vs 94.1%) and disease-free survival (88.2% vs 90.2% vs 83.0%).

**Conclusion:**

Our retrospective review suggests that splenic flexure colectomy in minimally invasive surgery is a safe and effective treatment option for splenic flexure colon cancer. The rate of metastatic lymph nodes at the root of the central artery and gastroepiploic arcade node was relatively low.

## Introduction

Colorectal cancer (CRC) is one of the most common gastrointestinal malignancies (1). Cancer of the splenic flexure is defined as colon cancer situated between the distal third of the transverse colon and the proximal descending colon within 10 cm of the flexure (2, 3). Splenic flexure cancer is relatively rare, representing only 2-8% of all colon cancers (4, 5). According to reports, splenic flexure cancer is associated with a poor prognosis due to a high risk of obstruction and diagnosis an advantage stage (2, 6). However, many studies have found no difference in survival compared to that of other colon cancers (4, 7).

No consensus has been reached regarding the optimal extent of radical surgery for splenic colon cancer. Lymphatic drainage is variable and incompletely understood in the splenic flexure. Various extents of resection have been proposed, from extended procedures to segmental resection. There is also some confusion regarding the definition of operations for splenic flexure cancer. Confusing nomenclature is often found in the literature (8). In an updated meta-analysis (9), a study concluded that the procedures include splenic flexure colectomy (SFC), left hemicolectomy (LHC), extended right hemicolectomy (ERHC) and subtotal colectomy (STC). Some authors believe that extended resection better guarantees the removal of all potentially included lymph nodes along the superior mesenteric vessels (2, 10, 11). In contrast, other authors reported that limited resection is sufficient and that results did not indicate a survival disadvantage (5, 12). Some surgeons have also recommended extended resection of the spleen and distal pancreas (3, 13), although the benefit of this approach is under debate (14, 15).

Moreover, the choice of operation is dependent on several factors, including the surgeon’s preference. One anonymous practice survey of members of the Association of Coloproctology of Great Britain and Ireland (ACPGBI) indicated that ERHC is preferred by 63% of respondents, followed by LHC (23%) and SFC (14%) (16). However, a more recent French intergroup survey showed that the preferred procedure was SFC (70%), followed by LHC (17%) and STC (13%) (17).

The objective of this study was to help improve surgical treatment for splenic flexure cancer. We compared the results of three different procedures (splenic flexure colectomy, left hemicolectomy and extended left hemicolectomy) with respect to postoperative, pathological and long-term results.

## Materials and Methods

### Patients

We retrospectively reviewed the data of patients who underwent radical surgery at Fujian Medical University Union Hospital from January 2011 to December 2018. Splenic flexure cancer was defined as a tumor located between the distal third of the transverse colon and the proximal descending colon within 10 cm of the flexure. Patients with metastasis, metachronous or synchronous colorectal cancers and familial adenomatous polyposis were excluded. We excluded patients who underwent palliative resection and/or emergency surgery because of perforation or acute obstruction. Finally, we retrieved 117 patients who underwent surgery for splenic flexure cancer.

The patient characteristics included age, sex, surgical approach, type of resection, operative details, histological results, postoperative outcomes and oncological follow-up results. The postoperative surgical outcomes included complications and mortality rates within 30 days of the surgery. The number of harvested lymph nodes and the presence of positive lymph nodes were recorded. Locoregional recurrence was defined as recurrence at the anastomosis or within the lymphatic drainage area in the region of the primary tumor. Distant recurrence was defined as recurrent tumors in the peritoneum, liver, nonregional lymph nodes or locations outside the abdominal cavity. The disease stage was evaluated according to the American Joint Committee on Cancer Staging, 7th Edition. The primary outcome was disease free survival. The second outcome was overall survival and postoperative outcomes.

### Surgical Techniques

Splenic flexure colectomy (SFC) was defined as resection of the distal part of the transverse colon and the descending colon by ligating the left colic artery (LCA) with or without ligating the left branch of the middle colic artery (MCA) or accessory middle colic artery (aMCA).

Left hemicolectomy (LHC) was defined as resection of the distal part of the transverse colon, the descending colon, and the sigmoid colon by ligating the MCA and LCA.

Extended left hemicolectomy (ELHC) was defined as resection of the distal part of the transverse colon, descending and sigmoid colon down to the rectosigmoid union by ligating the inferior mesenteric artery (IMA) with or without ligating other arteries.

D3 lymphadenectomy was routinely performed. In all the cases, the anastomosis type was selected according to the surgeon’s judgment. The extended resection was usually indicated with one or more of following conditions identified with the support of the radiologist at the preoperative evaluation: T4 stage, metastatic regional lymph nodes, clear vascular involvement, large tumor size. The lymph nodes were dissected from the fresh surgical specimen by surgeon.

Adjuvant chemotherapy was administered for stage III patients and stage II patients with a high risk of recurrence, except in cases of medical contraindications. All patients underwent routine follow-up every 3 months for the first 2 years and every 6 months thereafter. A physical examination, measurement of the CEA level, chest X-ray or CT, and abdominopelvic MRI or CT were performed at each visit. Colonoscopy was conducted annually after surgery.

### Statistical Analysis

Statistical analysis was performed using the SPSS software program (ver. 23 SPSS Inc., Chicago, IL, USA). Chi-square and Fisher’s tests were applied, as appropriate, to compare categorical variables. Quantitative data were compared using ANOVA. Survival rates were analyzed using the Kaplan–Meier method and compared with the log-rank test. A two-tailed P value <0.05 was considered significant statistically.

## Results

### Patient Characteristics and Perioperative Outcomes

Of the 117 patients, 73 (62.4%) underwent SFC, 22 (18.8%) underwent LHC, and 22 (18.8%) underwent ELHC. The mean age of the patient population was 58.1 years, and 59.8% were male. All three groups had similar sex, age and BMI distributions. No differences were found among the three groups in the baseline characteristics, as reported in **Table 1**. A total of 36.8% of patients were diagnosed with colonic stenosis caused by tumors.

**Table 1:** Demographics of patients treated by SFC, LHC or ELHC for splenic flexure cancer.

Characteristics	SFC	LHC	ELHC	*P* value
	(n=73)	(n=22)	(n=22)	
Sex				0.965
male	43 (59%)	13 (59%)	14 (64%)	
female	30 (41%)	9 (41%)	8 (36%)	
Age (year)	56.6 ± 14.5	59.8 ± 16.4	61.2 ± 14.6	0.378
BMI (kg/m^2^)	22.38 ± 3.2	21.90 ± 2.9	23.29 ± 3.2	0.314
Pre-operative hemoglobin (g/L)	120 ± 24.0	115 ± 25.9	123 ± 23.6	0.553
Albumin serum level (g/L)	38.7 ± 4.7	38.1 ± 5.0	40.6 ± 3.9	0.156
CEA (ng/ml)	8.6 ± 28.2	14.4 ± 21.0	9.1 ± 12.8	0.628
CA199 (u/ml)	38.2 ± 126.5	15.4 ± 11.1	57.96 ± 88.1	0.420
Presence of colonic stenosis				0.295
Yes	26 (35.6%)	6 (27.3%)	11 (50%)	
No	47 (64.4%)	16 (72.7%)	11 (50%)	

SFC, splenic flexure colectomy; LHC, left hemicolectomy; ELHC, extended left hemicolectomy; Pre-, preoperative.

A laparoscopic approach was adopted for 85.5% of patients. There was no significant difference among the groups in surgical approach or blood loss. The duration of the operation was significantly longer for the SFC procedure (P=0.003). Conversion to laparotomy was necessary in 3.8% of cases. Two conversions were due to uncontrolled hemorrhage in the spleen area and IMA. The overall rate of complications was 27.4% (32/117). Sever postoperative complications (Clavien III–V) occurred in 3 patients (2.6%). When analyzing the development of each complication, no differences were demonstrated among the groups (**Table 2**). All complications were resolved with conservative treatments. No reoperation or mortality were observed. No statistically significant difference was observed with respect to length of hospital stay among groups (P=0.684).

**Table 2:** Postoperative outcomes of patients treated by SFC, LHC or ELHC for splenic flexure cancer.

Characteristics	SFC	LHC	ELHC	*P*1 value	*P*2 value	*P*3value	*P* value
	(73)	(22)	(22)				
Type of approach				1.00	0.204	0.342	0.441
Laparotomy	9 (12.3%)	3 (13.6%)	1 (4.5%)				
Laparoscopic	62 (84.9%)	19 (86.4%)	19 (86.4%)				
Conversion	2 (2.7%)	0 (0%)	2 (9.1%)				
Operation time (minutes)	239.1 ± 62.1	222.7 ± 54.9	197.1 ± 42.6	0.269	0.004	0.091	0.012
Blood loss (ml),	83.6 ± 97.9	108.2 ± 79.7	88.6 ± 103.6	0.285	0.834	0.487	0.574
Postoperative complications				0.786	0.788	0.736	0.807
Yes	20 (27.4%)	5 (22.7%)	7 (31.8%)				
No	53 (72.6%)	17 (77.3%)	15 (68.2%)				
Clavien Score				1.00	0.551	1.00	0.627
1-2	71 (97.3%)	22 (100%)	21 (97.4%)				
3-4	2 (2.7%)	0 (0)	1 (2.6%)				
Anastomotic leakage				1.00	1.00	1.00	1.00
Yes	1 (1.4%)	0 (0%)	0 (0%)				
No	72 (98.6%)	22 (100%)	22 (100%)				
Anastomotic bleeding				1.00	0.232	1.00	0.384
Yes	0 (0%)	0 (0%)	1 (4.5%)				
No	73 (100%)	22 (100%)	21 (95.5%)				
Ileus				0.382	0.661	1.00	0.518
Yes	5 (6.8%)	3 (13.6%)	2 (9.1%)				
No	68 (93.2%)	19 (86.4%)	20 (90.9%)				
Chyle leak				0.570	1.00	1.00	0.822
Yes	4 (5.5%)	0 (0%)	1 (4.5%)				
No	69 (94.5%)	22 (100%)	21 (95.5%)				
Abdominal hemorrhage				1.00	0.411	1.00	0.602
Yes	1 (1.4%)	0 (0%)	1 (4.5%)				
No	72 (98.6%)	22 (100%)	21 (95.5%)				
Surgical site infection				0.330	1.00	0.488	0.571
Yes	6 (8.0%)	0 (0%)	2 (9.1%)				
No	67 (92.0%)	22 (100%)	20 (90.9%)				
Gastroparesis				0.411	1.00	1.00	0.606
Yes	1 (1.4%)	1 (4.5%)	0 (0%)				
No	72 (98.6%)	21 (95.5%)	22 (100%)				
Pneumonia				1.00	1.00	1.00	1.00
Yes	8 (11.0%)	2 (9.1%)	2 (9.1%)				
No	65 (89.0%)	20 (90.9%)	20 (90.9%)				
Length of stay (day)	9.2 ± 5.4	9.4 ± 4.5	8.2 ± 3.8	0.931	0.422	0.381	0.684

SFC, splenic flexure colectomy; LHC, left hemicolectomy; ELHC, extended left hemicolectomy.

P1: SFC vs LHC, P2: SFC vs ELHC, P3: LHC vs ELHC.

### Pathological Outcome

R0 resections were achieved in all patients. High or moderate grade tumors were present in 82.9% of cases. 12% of cases were mucinous carcinoma. pT4 tumors were found in 11.1% of cases. No differences were found among the three groups in terms of pTNM stage or lymphatic or venous invasion. The SFC group had a higher rate of nerve invasion than the LHC and ELHC groups (P=0.039). The mean number of harvested lymph nodes was 27.5 in the SFC group, 25.0 in the LHC group and 25.6 in the ELHC group (P=0.158). There was no difference among the groups regarding the number of metastatic lymph nodes removed. (**Table 3**).

**Table 3:** Pathological data.

Characteristics	SFC	LHC	ELHC	*P*1 value	*P*2 value	*P*3 value	*P* value
	(73)	(22)	(22)				
R0 resection	73 (100%)	22 (100%)	22 (100%)				
Differentiation				0.516	0.743	1.00	0.677
Well/moderately	62 (84.9%)	17 (77.3%)	18 (81.8%)				
Poorly	11 (15.1%)	5 (22.77%)	4 (18.2%)				
Mucinous histology				0.693	0.273	1.00	0.521
Yes	66 (90.4%)	19 (86.4%)	18 (81.8%)				
No	7 (9.6%)	3 (13.6%)	4 (18.2)				
pT stage				0.716	0.280	0.070	0.388
1	3 (4.1%)	0	1 (4.5%)				
2	4 (5.5%)	0	2 (9.1%)				
3	47 (64.4%)	15 (68.2%)	17 (77.3%)				
4	19 (26.0%)	7 (31.8%)	2 (9.1%)				
pN stage				0.107	0.931	0.255	0.319
0	44 (60.3%)	9 (40.9%)	14 (63.6%)				
1	23 (31.5%)	8 (36.4%)	6 (27.3%)				
2	6 (8.2%)	5 (22.7%)	2 (9.1%)				
pTNM stage				0.249	0.361	0.209	0.242
I	2 (2.7%)	0	2 (9.1%)				
II	42 (57.5%)	9 (40.9%)	12 (54.5%)				
III	29 (39.7%)	13 (59.1%)	8 (36.4%)				
Nerval invasion				0.019	0.552	0.233	0.039
Absent	58 (79.5%)	22 (100%)	19 (86.4%)				
Present	15 (20.5%)	0 (0%)	3 (13.6%)				
Lymphatic or vascular invasion				0.726	0.743	0.664	0.700
Absent	62 (84.9%)	20 (90.9%)	18 (81.8%)				
Present	11 (15.1%)	2 (9.1%)	4 (18.2%)				
Positive LN	1.0 ± 2.2	2.1 ± 2.6	1.1 ± 2.0	0.097	0.838	0.182	0.158
LN retrieved	27.5 ± 13.6	25.0 ± 10.3	25.6 ± 11.6	0.416	0.558	0.838	0.646

SFC, splenic flexure colectomy; LHC, left hemicolectomy; ELHC, extended left hemicolectomy.

LN, lymph nodes; P1: SFC vs LHC, P2: SFC vs ELHC, P3: LHC vs ELHC.

### Metastatic Lymph Node Distribution

**Figure 1:**
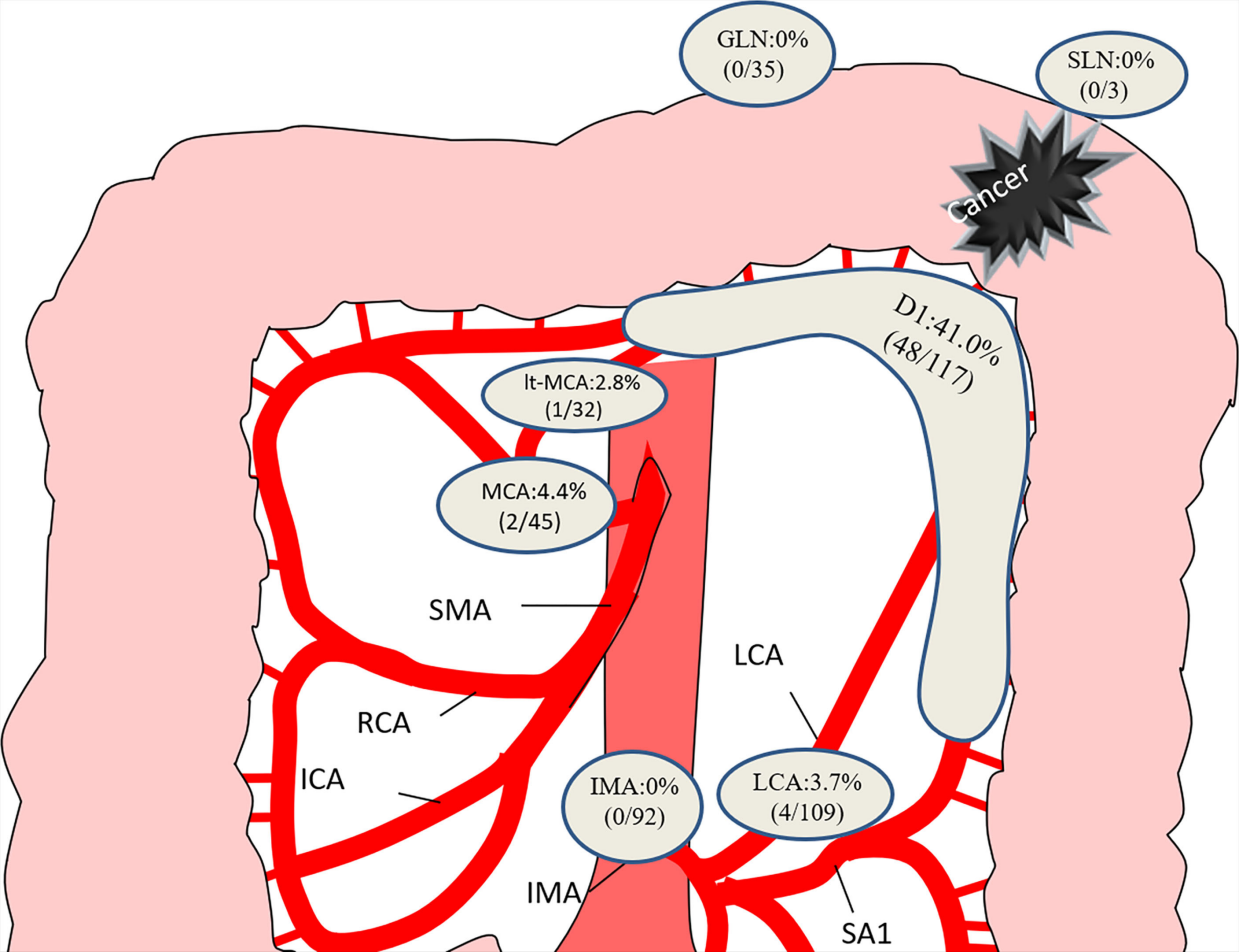
Distribution of lymph node metastasis in the 117 patients. GLN, gastroepiploic lymph node; SLN, splenic hilar lymph node; MCA, middle colic artery; LCA, left colic artery; IMA, inferior mesenteric artery.

The distribution of metastatic lymph nodes is shown in **Figure 1**. Paracolic lymph node metastasis developed in 41.0% (48/117) of patients. The rate of positive lymph nodes was similar between the LCA and the MCA. No metastasis was observed histologically in the gastroepiploic lymph nodes, splenic hilar lymph nodes, or lymph nodes at the root of the IMA.

### Long-Term Outcome

**Figure 2:**
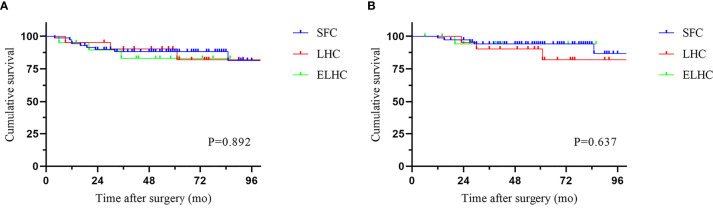
Survival according to surgical procedure. **(A)** Disease-free survival. **(B)** Overall survival.

**Figure 3:**
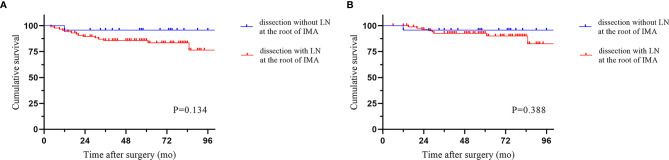
Survival according to whether lymph nodes at the root of the IMA were dissected. **(A)** Disease-free survival. **(B)** Overall survival.

**Figure 4:**
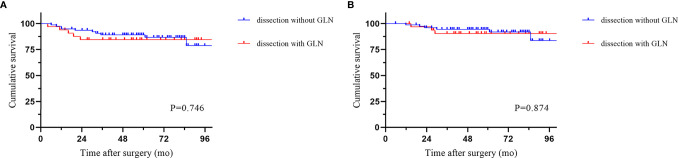
Survival according to whether gastroepiploic lymph nodes were dissected. **(A)** Disease-free survival. **(B)** Overall survival.

The median follow-up period was 58.5 months (IQR 6-113). Local recurrence was found in 4.3% of cases, and distant recurrence was found in 11.1% of cases. The recurrence patterns are reported in **Table 4**. There was no significant difference among the three groups in terms of recurrence. The cumulative 5-year DFS (88.2% vs 90.2% vs 83.0%) and OS (94.0% vs 90.2% vs 94.1%) rates were comparable among the three techniques (**Figure 2**). Furthermore, the survival analysis of patients who underwent dissection with or without removal of the lymph nodes at the root of the IMA did not show a significant difference (**Figure 3**). Dissection with or without removal of the gastroepiploic lymph nodes also showed similar outcomes in terms of DFS and OS (**Figure 4**).

**Table 4:** Long-term outcomes.

Characteristics	SFC	LHC	ELHC	*P* value
	(73)	(22)	(22)	
Anastomotic recurrence				1.00
No	67 (91.8%)	21 (95.5%)	20 (90.9%)	
Yes	2 (2.7%)	0 (0%)	0 (0)	
Missing	4 (5.5%)	1 (4.5%)	2 (9.1%)	
Locoregional recurrence				1.00
No	66 (90.4%)	20 (90.9%)	19 (86.4%)	
Yes	3 (4.1%)	1 (4.5%)	1 (4.5%)	
Missing	4 ( (5.5%))	1 (4.5%)	2 (9.1%)	
Distant recurrence				0.695
No	64 (87.7%)	19 (86.4%)	18 (81.8%)	
Yes	5 (6.8%)	2 (9.1%)	2 (9.1%)	
Missing	4 (5.5%)	1 (4.5%)	2 (9.1%)	

SFC, splenic flexure colectomy; LHC, left hemicolectomy; ELHC, extended left hemicolectomy.

## Discussion

Splenic flexure colon cancer was accounted for 2.0% of all colon cancer in our center. The present study showed that our surgical outcome and survival rate does not show significant differences in different surgical techniques for the treatment of cancers of this site. Our study supported the use of the SFC procedure.

Minimally invasive resection of splenic flexure colon cancer can be technically challenging. Michael et al. analyzed 842 patients reported that laparoscopy for splenic flexure colon cancer in carefully selected patients is associated with equivalent oncologic outcomes as well as improved short and long-term survival compared to an open approach (18). The conversion rate from laparoscopy to open surgery was 15.2% (18). In our study, the rate of laparoscopy for splenic flexure cancer was over 85%, and the conversion rate to laparotomy was low. In the literature, the postoperative complication rate and mortality rate for splenic flexure cancer were higher than those for cancers in other sites. Arévalo et al. (19) reported that the rate of postoperative complications was 50.6% and that the mortality rate was 6.47%. Binda et al. (20) concluded that 8.9% of patients underwent reoperation, and the postoperative mortality rate was 4.3%. The present study showed that postoperative complications were reported in 27.4% of patients. Neither reoperation nor mortality were observed. This might be related to the fact that this study did not include patients who underwent emergency surgery. No significant differences in complications were found among the groups. The longer duration of surgery in the SFC group may be because the procedure included dissection of the lymph nodes at the root of the IMA. Our retrospective review suggested that minimally invasive resection of splenic flexure colon cancers is a safe technique.

The lymphatic drainage pattern of splenic flexure cancer has not been completely elucidated. Theoretically, the lymphatic drainage pattern is consistent with the corresponding artery. Griffith (21) described that the splenic flexure is supplied by the terminal branches of the LCA in 89% of cases, and the left branch of the MCA was the supplying vessel in the remainder of the cases. Fukuoka et al. (22) reported six types of blood vessels supplying the splenic flexure: the MCA, aMCA, and LCA of the main feeder vessels. Vasey et al. (23)reported that lymphatic drainage of the splenic flexure was preferentially directed towards the LCA (96%) by using intraoperative scintigraphic mapping. Watanabe et al. (24) used indocyanine green fluorescence and revealed that in 61.3% of patients, lymph flow was directed to the area at the root of the inferior mesenteric vein (IMV).

Our results suggest that a splenic flexure colectomy is oncological adequate for splenic flexure colon cancer. In this study, we found no positive lymph nodes at the root of IMA, although lymph nodes can appear at the root of MCA, but in a low proportion. Nakagoe et al. (25)reported that the majority of lymph node metastases are located along the paracolic arcade, while no metastatic lymph nodes at the root of the MCA and IMA were observed. de’Angelis et al. (14) found no positive lymph nodes along the SMA. The above findings also supported the application of limited SFC. However, Manceau et al. (26)included 65 patients with splenic flexure cancer who underwent STC and found that positive lymph nodes were diagnosed in 9.2% of patients. However, we observed that the majority of distant lymph nodes were located along the RCA, and over 30% of did not have positive lymph nodes along the MCA, which was higher than previously reported. The author might misclassify the MCA or aMCA as the RCA. Of note, in the current study, we observed that patients with positive lymph nodes at the root of the MCA had no positive lymph nodes along the IMA. Patients with positive lymph nodes at the LCA were still alive without recurrence, even though none of them underwent dissection of the lymph nodes along the SMA. Watanabe et al. (24) found that no patients exhibited lymph flow in both the LCA and the left branch of the MCA. We supposed that the lymphatic drainage pattern of splenic flexure cancer might be mutually exclusive between the SMA and the IMA.

Perrakis et al. (13) recommended extended resection along with splenectomy and distal pancreatectomy due to potential lymphatic drainage. We found no metastatic lymph nodes along the gastroepiploic arcade or splenic hilum. We also observed no survival advantage from extended lymph node dissection.

Concerning oncological outcomes, R0 resection was achieved in all patients. The number of lymph nodes harvested has been identified as a surrogate marker of the quality of surgery (27). A minimum of 12 lymph nodes is currently accepted for correct staging (28). The proportion of patients with more than 12 lymph nodes harvested was not significantly different among the three groups and was over 90% in each group. With respect to long-term survival outcomes, the incidence of recurrence was relatively low regardless of the type of procedure. de’Angelis et al. (14)compared extended right colectomy versus left colectomy and found no differences between groups with respect to overall survival and disease-free survival. Beisani et al. (29) reported no difference in long-term oncological results between subtotal colectomy and left hemicolectomy, although more lymph nodes were harvested with subtotal colectomy. Rega et al. (30)reported that no difference in overall and progression free survival among the results of three different approaches (extended right hemicolectomy, extended left hemicolectomy and segmental resection). A network meta-analysis study reported no difference in OS regardless of the type of procedure, which ranged from splenic flexure colectomy to subtotal colectomy (9). Although the extent of resections and the inclusion criteria were different among the above studies, the results did not show a clear advantage in survival with extended resection.

The current study has some limitations mainly related to its retrospective nature, and the site of the lymph nodes might be misclassified. The study spans a long-time frame, and thus, historical bias cannot be excluded. This a monocentric retrospective study including just 117 patients, which is a quite small number even considering that SFC is rare.

## Conclusion

The results of this study showed that splenic flexure colon cancer was not associated with a worse prognosis. The rate of metastatic lymph nodes at the root of the central artery and gastroepiploic arcade node was relatively low. SFC, LHC and ELHC had the same postoperative, oncological, and survival outcomes. Therefore, splenic flexure colectomy in minimally invasive surgery is a safe and effective treatment option for splenic flexure colon cancer.

## Data Availability Statement

The original contributions presented in the study are included in the article/supplementary material. Further inquiries can be directed to the corresponding authors.

## Ethics Statement

The studies involving human participants were reviewed and approved by The Institutional Review Board of the Fujian Medical University Union Hospital. Written informed consent was obtained from the individual(s) for the publication of any potentially identifiable images or data included in this article.

## Author Contributions

MH, XW, and PC conceived and designed the study. MH, YS, XW, SH, and YH collated the data. MH, XW, and YS analyzed the data. MH, XW, and PC wrote the manuscript. All authors have read and approved the final version of this manuscript.

## Funding

This study was financially supported by the National Clinical Key Specialty Construction Project (General Surgery) of China (No. 2012-649), Natural Science Foundation of Fujian Province (2020J011030), Medical Science Research Foundation of Beijing Medical and Health Foundation (B20062DS), Medical innovation project of Fujian province (2020CXA025).

## Conflict of Interest

The authors declare that the research was conducted in the absence of any commercial or financial relationships that could be construed as a potential conflict of interest.

## Publisher’s Note

All claims expressed in this article are solely those of the authors and do not necessarily represent those of their affiliated organizations, or those of the publisher, the editors and the reviewers. Any product that may be evaluated in this article, or claim that may be made by its manufacturer, is not guaranteed or endorsed by the publisher.
